# KIR Polymorphisms Modulate Peptide-Dependent Binding to an MHC Class I Ligand with a Bw6 Motif

**DOI:** 10.1371/journal.ppat.1001316

**Published:** 2011-03-10

**Authors:** Arnaud D. Colantonio, Benjamin N. Bimber, William J. Neidermyer, R. Keith Reeves, Galit Alter, Marcus Altfeld, R. Paul Johnson, Mary Carrington, David H. O'Connor, David T. Evans

**Affiliations:** 1 Department of Microbiology and Molecular Genetics, Harvard Medical School, New England Primate Research Center, Southborough, Massachusetts, United States of America; 2 Department of Pathology and Laboratory Medicine, University of Wisconsin-Madison, Wisconsin National Primate Research Center, Madison, Wisconsin, United States of America; 3 Division of Immunology, New England Primate Research Center, Southborough, Massachusetts, United States of America; 4 Ragon Institute of Massachusetts General Hospital, Massachusetts Institute of Technology and Harvard University, Boston, Massachusetts, United States of America; 5 Cancer and Inflammation Program, Laboratory of Experimental Immunology, SAIC-Frederick, Inc., NCI Frederick, Frederick, Maryland, United States of America; NIH/NIAID, United States of America

## Abstract

Molecular interactions between killer immunoglobulin-like receptors (KIRs) and their MHC class I ligands play a central role in the regulation of natural killer (NK) cell responses to viral pathogens and tumors. Here we identify Mamu-A1*00201 (Mamu-A*02), a common MHC class I molecule in the rhesus macaque with a canonical Bw6 motif, as a ligand for Mamu-KIR3DL05. Mamu-A1*00201 tetramers folded with certain SIV peptides, but not others, directly stained primary NK cells and Jurkat cells expressing multiple allotypes of Mamu-KIR3DL05. Differences in binding avidity were associated with polymorphisms in the D0 and D1 domains of Mamu-KIR3DL05, whereas differences in peptide-selectivity mapped to the D1 domain. The reciprocal exchange of the third predicted MHC class I-contact loop of the D1 domain switched the specificity of two Mamu-KIR3DL05 allotypes for different Mamu-A1*00201-peptide complexes. Consistent with the function of an inhibitory KIR, incubation of lymphocytes from *Mamu-KIR3DL05^+^* macaques with target cells expressing Mamu-A1*00201 suppressed the degranulation of tetramer-positive NK cells. These observations reveal a previously unappreciated role for D1 polymorphisms in determining the selectivity of KIRs for MHC class I-bound peptides, and identify the first functional KIR-MHC class I interaction in the rhesus macaque. The modulation of KIR-MHC class I interactions by viral peptides has important implications to pathogenesis, since it suggests that the immunodeficiency viruses, and potentially other types of viruses and tumors, may acquire changes in epitopes that increase the affinity of certain MHC class I ligands for inhibitory KIRs to prevent the activation of specific NK cell subsets.

## Author Summary

NK cells provide an important first line of defense against infectious diseases and tumors by virtue of their ability to kill infected or malignant cells without prior sensitization. NK cell activation is regulated in part through interactions between KIRs expressed on the surface of NK cells and their MHC class I ligands on target cells. Here we identify Mamu-A1*00201 (Mamu-A*02), a common MHC class I molecule in the rhesus macaque, as a ligand for Mamu-KIR3DL05. We show that this interaction is peptide-dependent, since soluble Mamu-A1*00201 tetramers folded with certain SIV peptides, but not others, stained cells expressing Mamu-KIR3DL05. Differences in binding avidity were associated with polymorphisms in the D0 and D1 domains of Mamu-KIR3DL05, whereas differences in peptide-specificity mapped to the D1 domain. These observations reveal a previously unappreciated role for D1 polymorphisms in determining the selectivity of KIRs for MHC class I-bound peptides, and identify the first functional KIR-MHC class I interaction in the rhesus macaque. These observations suggest that SIV, and potentially also HIV-1, may acquire changes in epitopes that increase the avidity of MHC class I ligands for inhibitory KIRs as a mechanism of immune evasion to prevent the activation of certain NK cell subsets.

## Introduction

Natural killer (NK) cells are able to lyse infected or malignant cells without prior antigenic stimulation, and thus provide an important innate defense against infectious agents and tumors [1], [2]. NK cell activation in primates is regulated in part through interactions between the highly polymorphic killer immunoglobulin-like receptors (KIRs) expressed on NK cells and their MHC class I ligands on target cells [1], [2]. KIRs are type I integral membrane proteins with either two or three immunoglobulin (Ig)-like extracellular domains (2D or 3D) that transduce either inhibitory or activating signals via long (L) or short (S) cytoplasmic domains, respectively. Engagement of inhibitory KIRs by MHC class I molecules on healthy cells normally suppresses NK cell activation [1], [3], [4]. However, if these interactions are perturbed, for instance as a result of MHC class I downregulation by HIV-1 Nef [5], [6], or presentation of a peptide antagonist [7], this inhibition is lost resulting in NK cell activation and target cell lysis.

In contrast to the T cell receptor, which is highly specific for a given peptide-MHC complex, KIRs typically recognize subsets of MHC class I molecules with common amino acid motifs in their α1 domains. Based on serological epitopes that correspond to defined sequences at positions 77-83, all HLA-B molecules, and some HLA-A molecules, can be classified as either Bw4 or Bw6 allotypes [8]. Allotypes of KIR3DL1 have broad specificity for HLA-Bw4 ligands [9], whereas KIRs specific for HLA-Bw6 have not been identified. All inhibitory KIRs that have been examined thus far also exhibit selectivity for peptides bound by their MHC class I ligands [10], [11], [12], [13], [14], [15], [16]. These observations are consistent with crystal structures of KIR2DL1 and KIR2DL2 in complex with their HLA-C ligands showing that KIR residues contact surfaces of the HLA class I α1 and α2 domains in an orthogonal orientation across C-terminal residues of the bound peptide [17], [18]. However, the molecular basis for the selectivity of KIRs for different peptides bound by a particular MHC class I ligand has not been defined.

Genetic evidence suggests that polymorphic differences in the *KIR* and *HLA class I* genes play an important role in determining the course of infection for a number of human viral pathogens, including HIV-1 [19], [20], hepatitis C virus [21], human papillomavirus [22] and cytomegalovirus [23]. In the case of HIV-1, combinations of both activating and inhibitory *KIR3DL1/S1* and *HLA-Bw4* alleles have been associated with delayed progression to AIDS [19], [20]. NK cells expressing KIR3DS1 were also shown to suppress the *in vitro* replication of HIV-1 in target cells expressing HLA-Bw4 [24]. While these observations point to a role for KIR-MHC class I interactions in determining the outcome of HIV-1 infection, studies to address the functional significance of these interactions have been limited, in part, by the lack of a suitable animal model.

Simian immunodeficiency virus (SIV) infection of the rhesus macaque is an important animal model for lentiviral pathogenesis and for AIDS vaccine development [25]. Rhesus macaques express MHC class I molecules that correspond to products of the classical *HLA-A* and *-B* genes (*Macaca mulatta*; *Mamu-A* and *-B*), but not the *HLA-C* gene [26], [27]. Consistent with the co-evolution of KIR and MHC class I molecules, genes for the two-domain KIRs specific for HLA-C have not been identified in macaques [28], [29]. Instead, macaques have an expanded repertoire of *KIR3DL* genes characterized by extensive polymorphism and gene duplication [28], [29], [30], [31], [32].

Here we identify Mamu-A1*00201, a common rhesus macaque MHC class I molecule with a Bw6 motif, as a ligand for multiple allotypes of Mamu-KIR3DL05. We show that the binding of Mamu-KIR3DL05 to Mamu-A1*00201 is peptide-dependent, and that the relative avidity and peptide-selectivity of binding is determined by polymorphisms in the D0 and D1 domains. We also demonstrate that target cells expressing Mamu-A1*00201 suppress the degranulation of primary Mamu-KIR3DL05^+^ NK cells. These observations reveal a previously unappreciated role for D1 polymorphisms in determining the selective recognition of MHC class I-bound peptides by KIRs, and define the first functional KIR-MHC class I interaction in the rhesus macaque.

## Results

### Peptide-dependent tetramer staining of primary rhesus macaque NK cells

**Figure 1:**
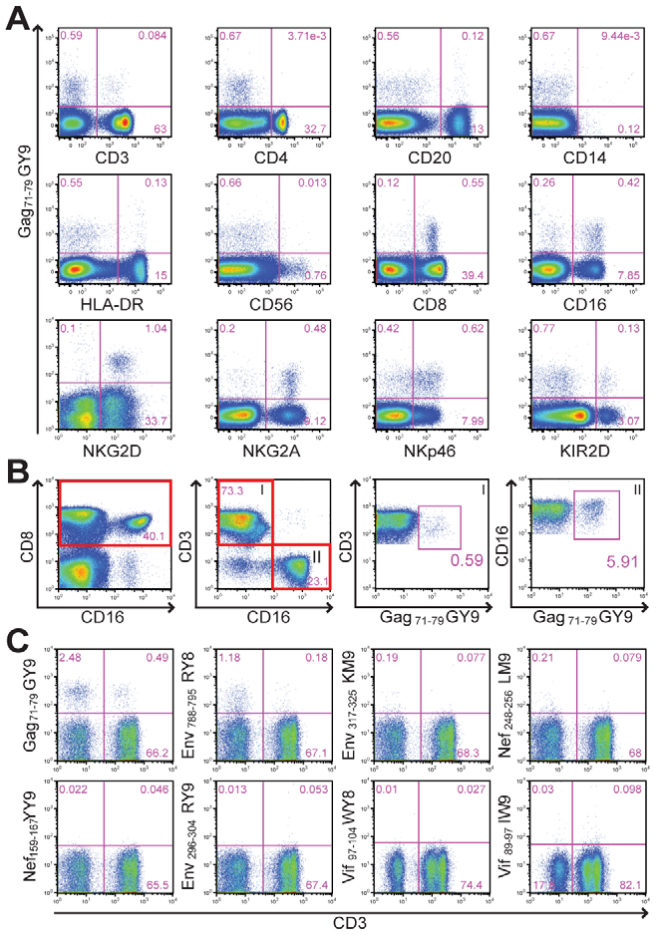
Peptide-dependent tetramer staining of primary NK cells and CD8^+^ T cells from an unimmunized, uninfected rhesus macaque. (A) To identify the tetramer-positive cells, whole blood was stained with Mamu-A1*00201 Gag_71-79_ GY9 followed by monoclonal antibodies to the indicated cell type-specific markers. (B) The frequency of tetramer-positive CD8^+^ T cells versus CD16^+^ NK cells was determined by gating sequentially on CD8 followed by CD3 (I) or CD16 (II). (C) To determine if tetramer staining is dependent on the peptide bound by Mamu-A1*00201, whole blood was stained with Mamu-A1*00201 tetramers folded with eight different SIV peptides, Gag_71-79_ GY9 (GSENLKSLY), Env_788-795_ RY8 (RTLLSRVY), Env_317-325_ KM9 (KTVLPVTIM), Nef_248-256_ LM9 (LTARGLLNM), Nef_159-167_ YY9 (YTSGPGIRY), Env_296-304_ RY9 (RTIISLNKY), Vif_97-104_ WY8 (WTDVTPNY), and Vif_89-97_ IW9 (ITWYSKNFW), followed by monoclonal antibodies to CD3, CD8 and CD16. After gating on CD8^+^ lymphocytes, the percentages of tetramer-positive CD3^–^ versus CD3^+^ cells were determined.

Samples of peripheral blood from Mamu-A1*00201^+^ rhesus macaques were stained with Mamu-A1*00201 tetramers folded with SIV peptides to establish baseline CD8^+^ T cell responses prior to beginning a vaccine study. To our surprise, Mamu-A1*00201 in complex with the Gag_71-79_ GY9 peptide stained a subset of CD8^+^CD3^–^ lymphocytes from one animal (Mm 337-07). Plasma from this animal tested negative for SIV RNA and for antibodies to viral antigens, indicating that this animal had not been previously exposed to SIV. The majority of tetramer-positive cells expressed CD8α and CD16, characteristic of NK cells that are capable of mediating cytolytic activity [33], as well as additional NK cell markers including NKp46, NKG2A, and NKG2D (Fig. 1A). A subset of these cells also cross-reacted with an antibody to human KIR2D (Fig. 1A). Although most of the tetramer-positive cells were CD16^+^CD3^–^ NK cells, staining was also observed for CD8^+^CD3^+^ T cells (Fig. 1B). A longitudinal comparison of the frequency of tetramer-positive CD8^+^CD3^+^ versus CD16^+^CD3^–^ lymphocytes revealed that these two populations were relatively stable in this animal over more than a year, ranging from 0.16% to 0.69% for CD8^+^ T cells and from 5.1% to 9.8% for CD16^+^ NK cells. To investigate the contribution of the peptide bound by Mamu-A1*00201 to this unusual pattern of tetramer staining, whole blood was stained with Mamu-A1*00201 tetramers folded with peptides corresponding to eight different CD8^+^ T cell epitopes of SIV [34]. In addition to Gag_71-79_ GY9, staining was also observed for Env_788-795_ RY8, but not for any of the other tetramers (Fig. 1C). Thus, the tetramer staining observed for primary NK cells and CD8^+^ T cells from Mm 337-07 was dependent on the peptide bound by Mamu-A1*00201.

### Identification of Mamu-KIR3DL05 as a receptor for Mamu-A1*00201

**Figure 2:**
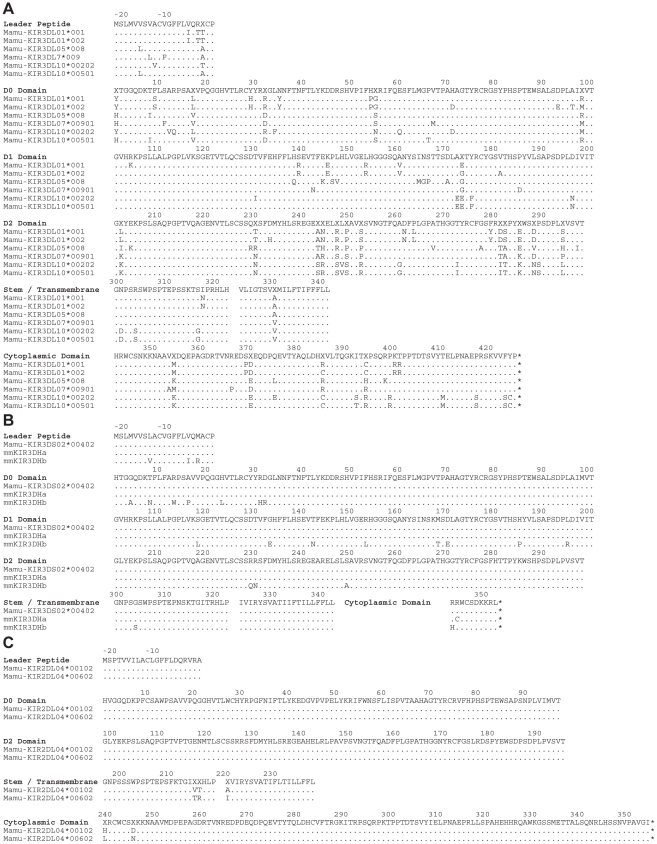
Amino acid sequence alignments for KIR alleles cloned from a rhesus macaque with a tetramer-positive NK cell population in peripheral blood. The predicted amino acid sequences are shown for six *Mamu-KIR3DL* alleles (A), three *Mamu-KIR3DS* alleles (B), and two *Mamu-KIR2DL04* alleles (C). Positions of amino acid identity with the consensus sequence shown at the top are indicated by a period and translational stop sites are indicated with an asterisk.

Since KIRs are known to be expressed on subsets of human NK cells and CD8^+^ T cells [4], [35], [36], we hypothesized that this pattern of tetramer staining might reflect Mamu-A1*00201 binding to a rhesus macaque KIR. Full-length *KIR* cDNA sequences were therefore cloned from the PBMCs of Mm 337-07 and sequenced. Six *KIR3DL* alleles, three *KIR3DS* alleles and two *KIR2DL04* alleles were identified in this animal, and their predicted amino acid sequences are shown in Fig. 2.

To identify the receptor for Mamu-A1*00201, Jurkat cells were transfected with constructs expressing each of the *KIR* alleles cloned from Mm 337-07 and stained with Mamu-A1*00201 tetramers. To differentiate transfected from untransfected cells, the *KIR* alleles were expressed from a bicistronic vector that co-expresses enhanced green fluorescent protein (eGFP). Since not all KIRs are well expressed on the cell surface, and antibodies are not available to macaque KIRs, an HA tag was introduced at the N-terminus of the D0 domain of each KIR. Our rationale for introducing the HA tag at this position is based on a recent three-dimensional model showing that the N-terminus of KIR3DL1 is free and oriented away from surfaces that are predicted to contact the peptide-MHC class I complex [37], and experiments demonstrating that the introduction of an epitope tag at the N-terminus of the D0 domain does not interfere with ligand recognition [38]. Following the electroporation of Jurkat cells with these KIR expression constructs, the cells were stained with Mamu-A1*00201 tetramers and with a monoclonal antibody to the HA tag. Transfected cells were identified by gating on the eGFP^+^ population, the surface expression of each KIR was verified by HA staining, and binding to Mamu-A1*00201 in complex with Gag_71-79_ GY9 versus Nef_159-167_ YY9 was assessed by tetramer staining.

**Figure 3:**
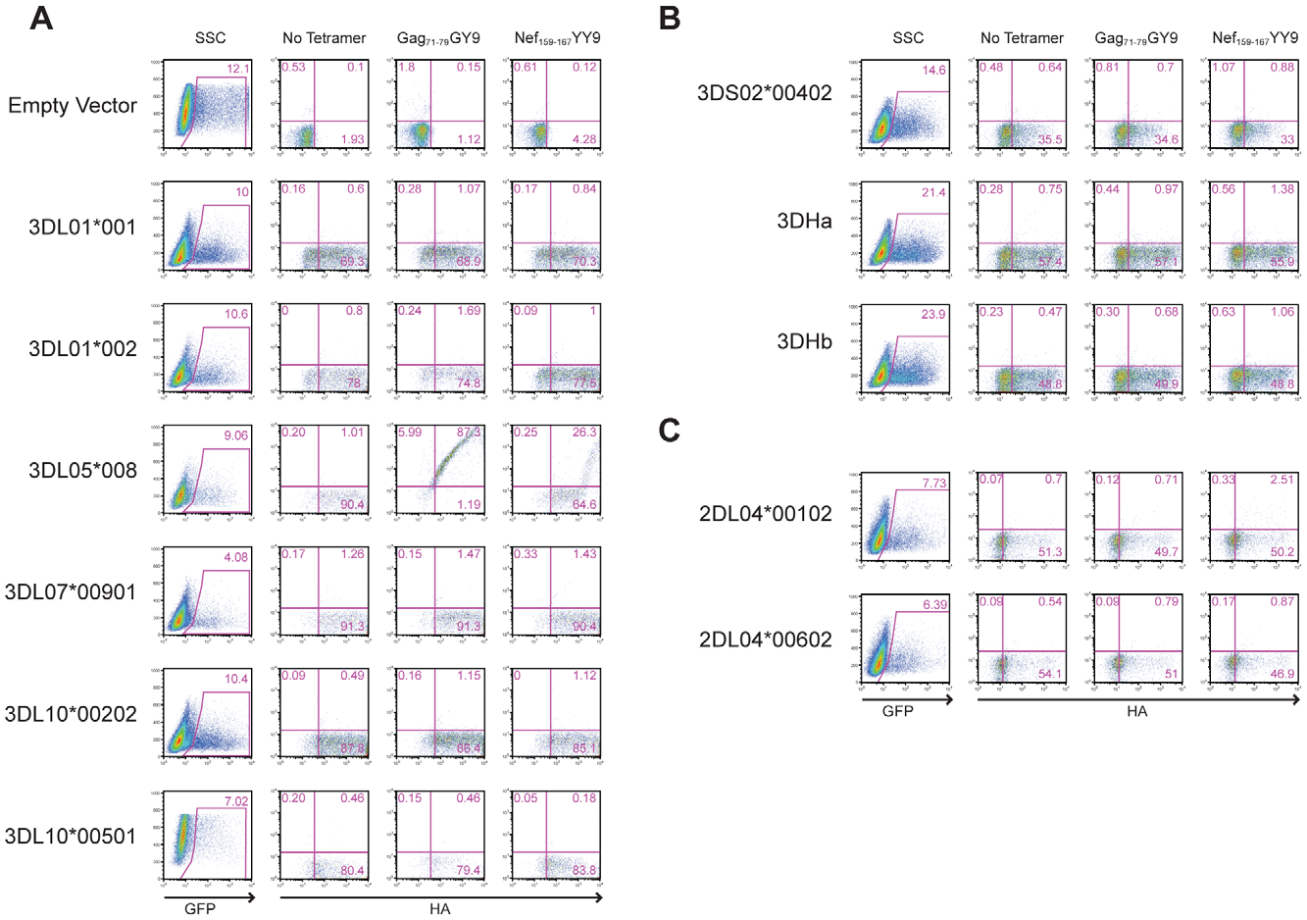
Mamu-A1*00201 is a ligand for Mamu-KIR3DL05. Jurkat cells were transfected with constructs expressing each of the six *Mamu-KIR3DL* (A), three *Mamu-KIR3DS* (B), and two *Mamu-KIR2DL04* (C) alleles cloned from Mm 337-07, and stained with Gag_71-79_ GY9 and Nef_159-167_ YY9 tetramers. The KIRs were expressed from a bicistronic vector designed to introduce a common leader peptide followed by an HA tag at the N-terminus of the D0 domain, and to co-express eGFP from a downstream internal ribosomal entry site. Jurkat cells were electroporated with the KIR expression constructs and stained the following day with APC-conjugated Mamu-A1*00201 tetramers, followed by a PE-conjugated antibody to the HA tag. Tetramer versus HA staining was determined after gating on the eGFP^+^ cell population. Quadrant gates were set using empty vector-transfected controls stained with tetramer and antibody to the HA tag.

All of the KIRs were expressed on the cell surface under the conditions of this assay, as indicated by HA staining (Fig. 3). However, only Mamu-KIR3DL05*008 resulted in a detectable level of staining with the Gag_71-79_ GY9 tetramer (Fig. 3A). At higher levels of surface expression, staining was also observed for Nef_159-167_ YY9, indicating that this tetramer can bind to Mamu-KIR3DL05*008 under conditions of protein over expression (Fig. 3A). These results identify Mamu-KIR3DL05*008 as a receptor for Mamu-A1*00201, and indicate that the peptide bound by Mamu-A1*00201 can modulate this interaction.

### Polymorphic differences among allotypes of Mamu-KIR3DL05 modulate the relative avidity and peptide-selectivity of binding to Mamu-A1*00201

Phylogenetic comparisons of macaque *KIR3DL* sequences revealed that Mamu-KIR3DL05*008 belongs to a group of similar alleles found in both rhesus and cynomolgus macaques [29]. To determine if other allotypes of Mamu-KIR3DL05 could also bind to Mamu-A1*00201, Jurkat cells were transfected with constructs expressing six additional *Mamu-KIR3DL05* alleles, as well as six *Mamu-KIR3DL07* alleles. The transfected cells were then stained with Mamu-A1*00201 tetramers folded with four different SIV peptides to assess binding; Gag_71-79_ GY9, Env_788-795_ RY8, Nef_159-167_ YY9 and Vif_89-97_ IW9.

**Figure 4:**
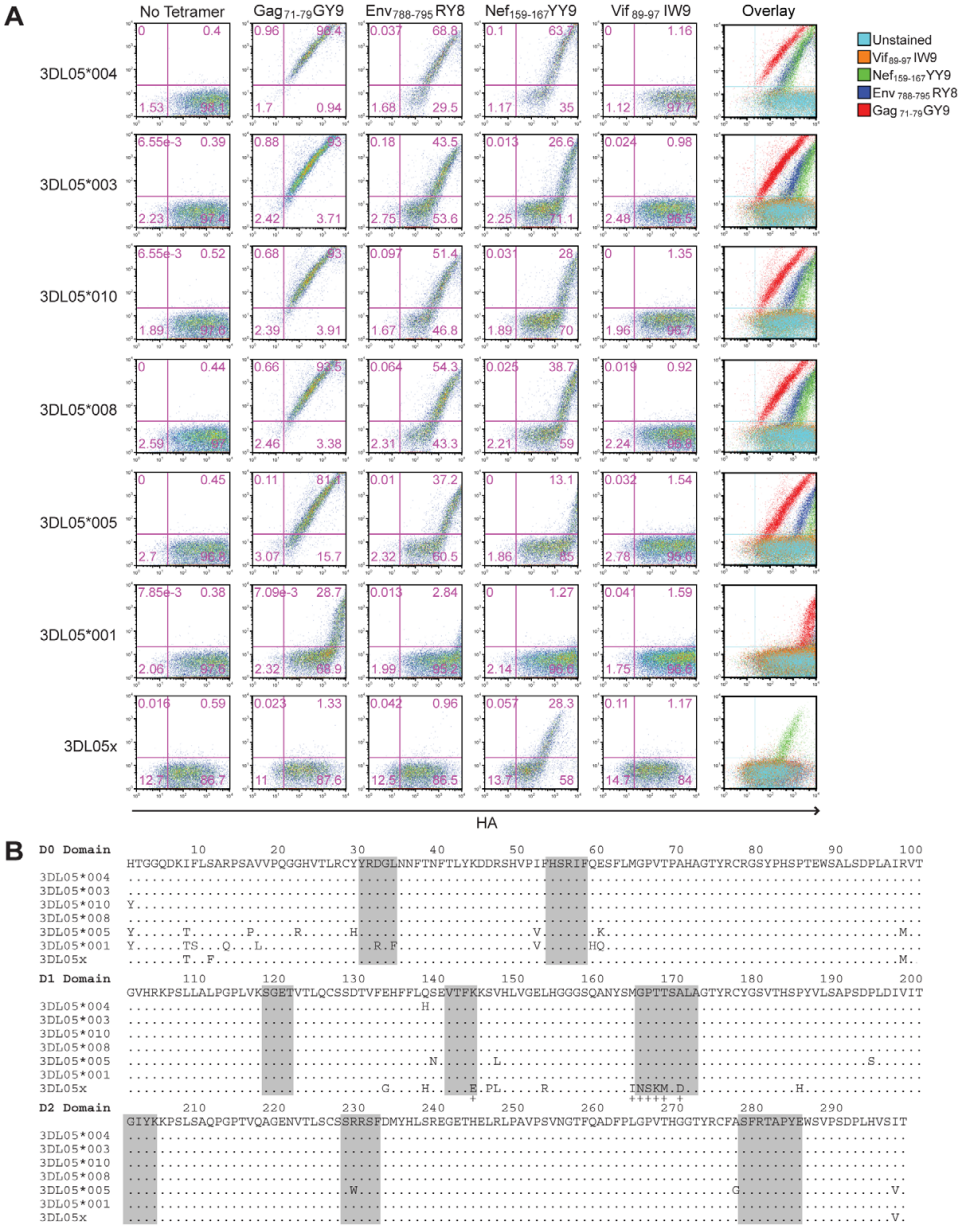
Polymorphisms in the D0 and D1 domains of Mamu-KIR3DL05 modulate the avidity and peptide-selectivity of binding to Mamu-A1*00201. (A) Jurkat cells were transfected with HA-tagged Mamu-KIR3DL05 expression constructs, and stained the next day with APC-conjugated Mamu-A1*00201 tetramers (Gag_71-79_ GY9, Env_788-795_ RY8, Nef_159-167_ YY9 or Vif_89-97_ IW9), followed by a PE-conjugated antibody to the HA tag. Quadrant gates were set using empty vector-transfected controls stained with tetramer and antibody to the HA tag. (B) An alignment comparing the predicted amino acid sequences of the D0, D1 and D2 domains for seven different *Mamu-KIR3DL05* alleles. Positions of amino acid identity with the consensus sequence are indicated by a period. The shaded regions correspond to loops predicted to contact surfaces of the peptide-MHC class I complex [37]. The plus signs beneath the alignment indicate unique residues in the D1 domain of mmKIR3DL05x that coincide with, or are immediately adjacent to, predicted MHC class I-contact loops.

One, or more, of the Mamu-A1*00201 tetramers bound to cells expressing each of the *Mamu-KIR3DL05* alleles. Cells expressing Mamu-KIR3DL05*004, -KIR3DL05*003, -KIR3DL05*010, -KIR3DL05*008 and -KIR3DL05*005 stained with Gag_71-79_ GY9, Env_788-795_ RY8 and Nef_159-167_ YY9, whereas cells expressing Mamu-KIR3DL05*001 and mmKIR3DL05x stained only with Gag_71-79_ GY9 or Nef_159-167_ YY9 (Fig. 4A). In contrast, none of these KIRs bound to the Vif_89-97_ IW9 tetramer (Fig. 4A). Furthermore, none of the *Mamu-KIR3DL07* alleles resulted in a detectable level of staining for any of the Mamu-A1*00201 tetramers (Fig. S1). Hence, this interaction is dependent on the peptide bound by Mamu-A1*00201 and is specific for Mamu-KIR3DL05.

Mamu-KIR3DL05*003, -KIR3DL05*008 and -KIR3DL05*010, were indistinguishable in their pattern of tetramer staining (Fig. 4A). This is reflected by the similarity in their values for the mean fluorescence intensity (MFI) of tetramer staining divided by the MFI of HA staining, which are provided in Table 1 as a quantitative comparison of tetramer binding corrected for differences in surface expression for each KIR. In accordance with the rank order of tetramer staining observed for primary NK cells (Fig. 1C), staining was highest for Gag_71-79_ GY9, followed by Env_788-795_ RY8, and then Nef_159-167_ YY9 (Fig. 4A and Table 1). With the exception of a single amino acid difference in the first position of the D0 domain of Mamu-KIR3DL05*010, each of these KIRs have identical Ig-like domains (Fig. 4B).

**Table 1:** Relative binding of Mamu-A1*00201 tetramers folded with four different SIV peptides to seven allotypes of Mamu-KIR3DL05.

	Mamu-A1*00201 tetramer			
Mamu-KIR3DL05	Gag_71-79_ GY9	Env_788-795_ RY8	Nef_159-167_ YY9	Vif_89-97_ IW9
Mamu-KIR3DL05*004	1.99	0.14	0.13	—
Mamu-KIR3DL05*003	1.57	0.06	0.03	—
Mamu-KIR3DL05*010	1.55	0.07	0.03	—
Mamu-KIR3DL05*008	1.49	0.07	0.03	—
Mamu-KIR3DL05*005	0.55	0.03	0.01	—
Mamu-KIR3DL05*001	0.02	—	—	—
mmKIR3DL05x	—	—	0.10	—

Jurkat cells were transfected with constructs expressing HA-tagged allotypes of Mamu-KIR3DL05 and stained the next day with one of the following Mamu-A1*00201 tetramers; Gag_71-79_ GY9, Env_788-795_ RY8, Nef_159-167_ YY9 or Vif_89-97_ IW9. The cells were then stained with a monoclonal antibody to the HA tag and analyzed by flow cytometry. Values represent the MFI of tetramer staining divided by the MFI of HA staining for the transfected, eGFP^+^ cell population. Values were not calculated if the number of tetramer-positive events was less than 1000.

Relative to Mamu-KIR3DL05*008, Mamu-KIR3DL05*004 exhibited an increase in the intensity of tetramer staining for Gag_71-79_ GY9 (1.3 fold), Env_788-795_ RY8 (2.0 fold) and Nef_159-167_ YY9 (4.3 fold) (Fig. 4A and Table 1). Since the Ig-like domains of Mamu-KIR3DL05*004 and Mamu-KIR3DL05*008 only differ by a single amino acid at position 138 (Fig. 4B), the histidine residue at this position accounts for the increase in Mamu-KIR3DL05*004 binding to Mamu-A1*00201. Based on a recently proposed three-dimensional model of KIR3DL1*015 bound to HLA-A*2402 [37], this residue is predicted to lie at the base of the second MHC class I-contact loop of the D1 domain, and may alter the conformation of this loop in a way that enhances binding to Mamu-A1*00201.

Compared to Mamu-KIR3DL05*008, decreases in the intensity of tetramer staining were observed for both Mamu-KIR3DL05*005 and -KIR3DL05*001 (Fig. 4A). The intensity of staining for Mamu-KIR3DL05*005, which differs from Mamu-KIR3DL05*008 by 14 amino acids (Fig. 4B), was 2.7-fold lower for Gag_71-79_ GY9, 2.3-fold lower for Env_788-795_ RY8 and 3.0-fold lower for Nef_159-167_ YY9 (Table 1). A much greater reduction in the intensity of tetramer staining was observed for Mamu-KIR3DL05*001. Tetramer staining for Mamu-KIR3DL05*001 was only detectable with Gag_71-79_ GY9 at an intensity that was 75-fold lower than for Mamu-KIR3DL05*008 (Table 1). Since Mamu-KIR3DL05*001 and -KIR3DL05*008 differ by ten amino acids in D0, but are otherwise identical in D1 and D2 (Fig. 4B), this reduction in the avidity of binding to Mamu-A1*00201 is due to polymorphic differences in the D0 domain. Thus, similar to KIR3DL1-HLA-Bw4 interactions in humans [37], [39], polymorphisms in the D0 domain of Mamu-KIR3DL05 can dramatically affect binding to MHC class I ligands.

In the case of mmKIR3DL05x, tetramer staining was observed for Nef_159-167_ YY9, but not for Gag_71-79_ GY9 or Env_788-795_ RY8 (Fig. 4A). This shift in the pattern of Mamu-A1*00201 tetramer staining almost certainly reflects differences in D1, since mmKIR3DL05x has a unique D1 domain, but nearly identical D0 and D2 domains to other allotypes of Mamu-KIR3DL05 (Fig. 4B). Using cryopreserved PBMCs from the original source of *mmKIR3DL05x*, we verified that *mmKIR3DL05x* represents a *bona fide* allele, and not a PCR artifact, by independently cloning and confirming the cDNA sequence for this allele, and by PCR amplification of a 2.0 kb region spanning intron 4 from genomic DNA with primers to unique sequences in exons 4 and 5. Additional sequence comparisons revealed that the D1 domain of mmKIR3DL05x, as well as the leader peptide and the D0 domain, are identical to Mamu-KIR3DS02*00402 and mmKIR3DHa (Fig. S2). Thus, *mmKIR3DL05x* appears to be the product of a recombination event in which exon 4 (encoding D1) was acquired, either by the introduction of exons 1-4 of a *Mamu-KIR3DS* gene into -*KIR3DL05* or by the introduction of exons 5-9 of *Mamu-KIR3DL05* into a *-KIR3DS* gene.

**Figure 5:**
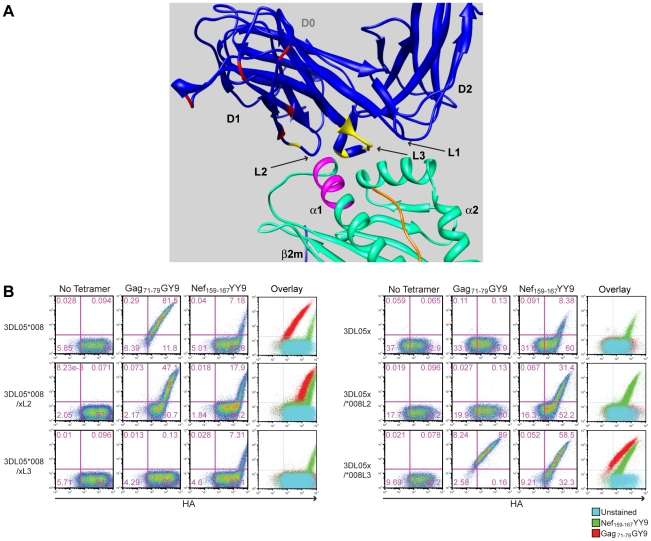
Amino acid differences in the third MHC class I-contact loop of the D1 domain account for the preferential binding of mmKIR3DL05x to the Nef_159-167_ YY9 tetramer. (A) Positions in the D1 domain of mmKIR3DL05x that differ from Mamu-KIR3DL05*008 are highlighted in a three-dimensional model of KIR3DL*015 bound to HLA-A*2402 [37]. The residues indicated in yellow are located in surface-exposed loops in close proximity to the bound peptide. The residues indicated in red represent differences in the D1 domain at sites that do not contribute directly to interactions with MHC class I ligands. The residues highlighted in magenta represent positions 77-83 of the α1 domain corresponding to the Bw6 of Mamu-A1*00201. (B) Jurkat cells were electroporated with constructs expressing recombinants of Mamu-KIR3DL05*008 and mmKIR3DL05x, for which residues of the second (L2) and third (L3) predicted MHC class I-contact loops in D1 were exchanged, and stained with APC-conjugated tetramer (Gag_71-79_ GY9 or Nef_159-167_ YY9) followed by a PE-conjugated antibody to the HA tag. Tetramer versus HA staining was determined after gating on the eGFP^+^ cell population and quadrant gates were set using control cells transfected with an empty vector.

A closer examination of mmKIR3DL05x revealed that seven of the thirteen differences in the D1 domain coincide with, or are immediately adjacent to, loops predicted to contact surfaces of the peptide-MHC class I complex [37]. These include a charge difference at position 144 in the second loop (L2) and a cluster of six residues at positions 164–170 in the third loop (L3) (Fig. 4B and Fig. 5A). To determine if these differences account for the unique binding pattern exhibited by mmKIR3DL05x, we constructed recombinants in which these sequences were exchanged with the corresponding sequences of Mamu-KIR3DL05*008, and tested them for binding to Gag_71-79_ GY9 versus Nef_159-167_ YY9. Reciprocal L2 substitutions affected the avidity, but not the specificity, of tetramer binding (Fig. 5B). In contrast, exchanging L3 residues switched the specificity, and altered the avidity, of binding to the Mamu-A1*00201 tetramers. The 3DL05*008/xL3 recombinant bound Nef_159-167_ YY9, but not Gag_71-79_ GY9, and the 3DL05x/*008L3 recombinant bound both Gag_71-79_ GY9 and Nef_159-167_ YY9 (Fig. 5B). Hence, these results reveal a role for polymorphisms in the third predicted contact loop of the D1 domain in determining the selective recognition of different peptides bound by the same MHC class I molecule.

### Mamu-A1*00201 suppresses the activation of tetramer-positive NK cells from Mamu-KIR3DL05^+^ macaques

**Figure 6:**
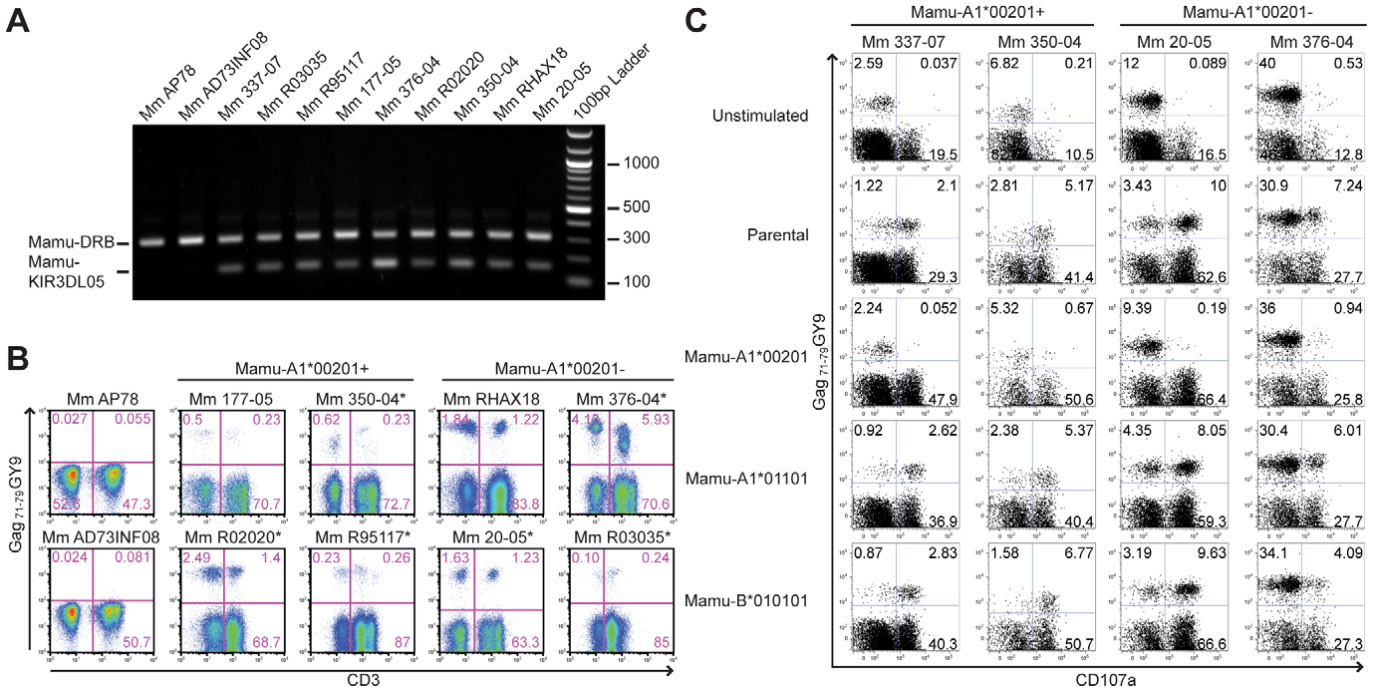
Mamu-A1*00201^+^ target cells suppress the degranulation of tetramer-positive NK cells. (A) Primers specific for exon 5 of *Mamu-KIR3DL05* were used to amplify a 156 bp sequence from genomic DNA. Primers specific for a conserved 300 bp region of *Mamu-DRB* were included as an internal control. PCR products were separated on a 1% agarose gel containing ethidium bromide and visualized by UV transillumination. (B) Peripheral blood from two *Mamu-KIR3DL05^−^* and eight *Mamu-KIR3DL05^+^* rhesus macaques was stained with the Gag_71-79_ GY9 and monoclonal antibodies to CD3, CD8 and CD16. The percentages of tetramer-positive cells were determined for CD3^-^CD8^+^ versus CD3^+^CD8^+^ lymphocytes. Of the eight *Mamu-KIR3DL05^+^* animals, four were Mamu-A1*00201^+^ (Mm 177-05, Mm 350-04, Mm R02020 and Mm R95117) and four were Mamu-A1*00201^-^ (Mm 20-05, Mm 376-04, Mm RHAX18 and Mm R03035). With the exception of Mm AP78 and Mm AD73INF08, which were negative for *Mamu-KIR3DL05*, tetramer-positive NK cells were detected in peripheral blood for each of these animals. Mm AP78, Mm AD73INF08, Mm 177-05, and Mm RHAX18 were uninfected at the time of this analysis. Mm R02020 and Mm R95117 were infected with SIV_smm_E660. Mm 350-04 and 376-04 were infected with SIV_mac_239Δnef. Mm 20-05 and Mm R03035 were infected with SIV_mac_239. The SIV-infected animals are indicated with an asterisk. (C) Freshly isolated PBMC from two Mamu-A1*00201^+^ and two Mamu-A1*00201^-^ macaques were stimulated with parental 721.221 cells, or 721.221 cells expressing individual rhesus macaque MHC class I molecules. Mm 337-07 was uninfected at the time of this analysis. The cells were incubated overnight at a 5:1 PBMC to target cell ratio in the presence of a monoclonal antibody to CD107a. Following stimulation, the cells were stained with the Gag_71-79_ GY9 tetramer and antibodies to CD3, CD8, CD16 and NKG2A. After gating on CD3^-^NK2GA^+^ lymphocytes, the upregulation of CD107a on tetramer-positive versus tetramer-negative NK cells was determined.

Additional *Mamu-KIR3DL05^+^* rhesus macaques were identified by sequence-specific PCR and screened for tetramer-positive NK cells and CD8^+^ T cells. The Gag_71-79_ GY9 tetramer stained subsets of CD8^+^CD3^–^ and CD8^+^CD3^+^ lymphocytes in in peripheral blood from each of the *Mamu-KIR3DL05^+^* animals, but not from *Mamu-KIR3DL05^-^* animals (Fig. 6A and 6B). In accordance with the complex regulation of KIR expression, which is influenced by a number of factors including differences in gene content on different *KIR* haplotypes, differences in the repertoire of *MHC class I* genes and polymorphic differences in *KIR* genes [40], [41], [42], [43], there was considerable animal-to-animal variation in the frequency and intensity of tetramer staining (Fig. 6B). Variation in the frequency of tetramer-positive CD8^+^CD3^+^ lymphocytes, particularly in Mamu-A1*00201^−^ animals that do not have Gag_71-79_-specific CD8^+^ T cells, may also reflect changes in KIR expression on memory CD8^+^ T cells related to age and/or prior exposure to infectious agents, since similar changes have been associated with age and encounters with viral pathogens in humans [36], [44], [45]. Overall, these results demonstrate that the presence of the *Mamu-KIR3DL05* gene is predictive of Gag_71-79_ GY9 staining in peripheral blood, that this pattern of tetramer staining is independent of Mamu-A1*00201 and SIV infection, and that the variability of staining is typical of the heterogeneity of KIR expression on human NK cells and CD8^+^ T cells [36], [46].

The role of NK cells and CD8^+^ T cells that express Mamu-KIR3DL05 in SIV-infected animals remains to be determined. However, among the eight animals represented in Fig. 6B, there were no obvious differences in the percentage of tetramer-positive cells for either population that could be attributed to the status of SIV infection. Indeed, of the two uninfected animals (Mm 177-05 and Mm RHAX18), the two animals infected with attenuated SIV_mac_239 Δ*nef* (Mm 350-04 and Mm 376-04), and the two animals infected with pathogenic SIV_mac_239 (Mm R03035 and Mm 20-05), each pair had among the lowest and the highest frequencies of tetramer-positive lymphocytes (Fig. 6B). Some of the tetramer-positive CD8^+^CD3^+^ lymphocytes in the Mamu-A1*00201^+^ animals probably represent virus-specific CD8^+^ T cells, since we cannot differentiate binding of the Gag_71-79_ GY9 tetramer to Mamu-KIR3DL05 versus the T cell receptor. Nevertheless, in two of the three SIV-infected animals (Mm 350-04 and Mm R02020), the percentage of tetramer-positive cells was actually higher for the CD8^+^CD3^−^ population than for the CD8^+^CD3^+^ population (Fig. 6B). Although the explanation for this is presently unclear, it is possible that Mamu-KIR3DL05 interactions with Mamu-A1*00201 may suppress CD8^+^ T cell responses to the Gag_71-79_ GY9 epitope in *Mamu-KIR3DL05^+^* animals, which could explain the inconsistent, and often weak, CD8^+^ T cell responses to Gag_71-79_ GY9 that we and others have observed in SIV-infected, Mamu-A1*00201^+^ macaques.

To investigate the functional consequences of NK cell recognition of Mamu-A1*00201, PBMC from four *Mamu-KIR3DL05^+^* animals were stimulated with the MHC class I-deficient 721.221 cell line [47], or with 721.221 cells expressing either Mamu-A1*00201, -A1*01101 (Mamu-A*11) or -B*010101 (Mamu-B*01), and stained for CD107a as a degranulation marker. The cells were also stained with Gag_71-79_ GY9 to differentiate Mamu-KIR3DL05^+^ NK cells from Mamu-KIR3DL05^−^ NK cells. CD107a was upregulated on the surface of both tetramer-positive and tetramer-negative NK cells in response to parental 721.221 cells and 721.221 cells expressing Mamu-A1*01101 or -B*010101 (Fig. 6C). In contrast, CD107a was suppressed on tetramer-positive NK cells, but not on tetramer-negative NK cells, in the presence of target cells expressing Mamu-A1*00201 (Fig. 6C). The same pattern of NK cell activation/inhibition was also observed by intracellular cytokine staining for IFNγ (Fig. S3). Moreover, CD107a was suppressed on tetramer-positive NK cells from both Mamu-A1*00201^+^ and -A1*00201^−^ animals (Fig. 6C), indicating that these cells were responsive to Mamu-A1*00201 whether or not they were educated in the presence of this MHC class I molecule. These results are therefore consistent with the functional inhibition of Mamu-KIR3DL05^+^ NK cells by Mamu-A1*00201.

## Discussion

Polymorphic differences in the *KIR* and *HLA class I* genes play an important role in determining the course of infection for HIV-1 and for a number of other viral pathogens [19], [20], [21], [22], [23], [24]. However, studies to address the functional significance of KIR-MHC class I interactions have been hampered by the lack of a suitable animal model. In the present study, we identify Mamu-A1*00201, an MHC class I molecule present in approximately 20% of Indian origin rhesus macaques [48], as a ligand for multiple allotypes of Mamu-KIR3DL05. Although the frequency of specific alleles of *Mamu-KIR3DL05* remains to be determined, the *Mamu-KIR3DL05* gene was present in 42% of the rhesus macaques (43 of 103 animals) recently screened at the New England Primate Research Center by sequence-specific PCR. This suggests that animals expressing both Mamu-KIR3DL05 and -A1*00201 are sufficiently common for use in future studies to investigate the functional implications of this interaction with respect to the pathogenesis of SIV infection.

Genotyping for *Mamu-KIR3DL05* was predictive of Mamu-A1*00201 tetramer staining for primary NK cells and CD8^+^ T cells in peripheral blood. The pattern of staining observed for subsets of CD8^+^CD3^−^ and CD8^+^CD3^+^ lymphocytes from *Mamu-KIR3DL05* ^+^ animals, but not from *Mamu-KIR3DL05* ^−^ animals, is consistent with the variegated expression of KIRs on human NK cells and CD8^+^ T cells [4], [35], [36], [49], [50], [51]. Tetramer staining was independent of Mamu-A1*00201, reflecting the segregation of *KIR* and *MHC class I* genes on different chromosomes, and was detectable regardless of the status of SIV infection. Moreover, the variability in the frequency and intensity of tetramer staining among *Mamu-KIR3DL05* ^+^ animals was typical of the heterogeneity of KIR expression on human NK cells and CD8^+^ T cells [36], [46]. Although tetramer staining has been reported for NK cell clones and for transfected cells expressing human KIRs [11], [12], to our knowledge this is the first report of direct *ex vivo* tetramer staining of primary NK cells.

Incubation of peripheral blood lymphocytes from *Mamu-KIR3DL05^+^* macaques with target cells expressing Mamu-A1*00201 specifically suppressed the degranulation of tetramer-positive NK cells. These results are consistent with the functional inhibition of primary NK cells expressing Mamu-KIR3DL05 by Mamu-A1*00201. Furthermore, this inhibition was observed for tetramer-positive NK cells from Mamu-A1*00201^−^ as well as from Mamu-A1*00201^+^animals, indicating that these cells were responsive to Mamu-A1*00201, whether or not they were educated in animals that express this ligand. Although the mechanisms of NK cell education are not fully understood [52], there is evidence that the maturation of NK cells expressing inhibitory KIRs is dependent on interactions with self-MHC class I molecules, and that NK cells expressing a particular inhibitory KIR in the absence of an appropriate MHC class I ligand are rendered hyporesponsive [3], [53], [54]. Thus, the *in vitro* suppression of tetramer-positive NK cells from Mamu-A1*00201^−^ animals by target cells expressing Mamu-A1*00201 implies that these cells were educated for recognition of another MHC class I ligand. This is perhaps not surprising given the complexity of the rhesus macaque MHC class I genes [55], [56], and the ability of KIRs to recognize multiple MHC class I ligands with common amino acid motifs in their α1 domains [9], [57].

Based on haplotype modeling and phylogenetic comparisons, *Mamu-KIR3DL05* is predicted to represent a single genetic locus [29]. Although *KIR3DL05* is not orthologous to any of the human *KIR* genes, interactions between Mamu-KIR3DL05 and Mamu-A1*00201 resemble features of KIR3DL1 binding to HLA-Bw4. A three-dimensional model of KIR3DL1*015 bound to HLA-A*2402 was recently constructed based on a crystal structure of KIR2DL1 in complex with HLA-C*04 [17], [37]. This model predicts that surface-exposed loops in each of the three Ig-like domains of KIR3DL1 contact the HLA class I molecule over the C-terminus of the bound peptide, and that the specificity of KIR3DL1 for HLA-Bw4 is dependent on a salt bridge between glutamate 282 in the D2 domain of KIR3DL1 and arginine 83 in the α1 domain of HLA-Bw4 [37]. Consistent with this model, polymorphisms in the Ig-like domains of Mamu-KIR3DL05 were associated with differences in binding to Mamu-A1*00201.

Amino acid differences in D0 affected the relative avidity of Mamu-KIR3DL05 binding to Mamu-A1*00201. Compared to Mamu-KIR3DL05*003/*008, tetramer staining was diminished for both Mamu-KIR3DL05*001 and -KIR3DL05*005, which differ by eight and ten residues in D0 respectively. In the case of Mamu-KIR3DL05*001, which is otherwise identical to Mamu-KIR3DL05*003/*008 in D1 and D2, binding to Mamu-A1*00201 was all but eliminated. These results are analogous to previous observations showing that polymorphisms in the D0 domain of KIR3DL1 modulate the avidity of binding to HLA-Bw4 ligands [37], [39].

Polymorphisms in D1 altered the selective binding of Mamu-KIR3DL05 to Mamu-A1*00201 in complex with different SIV peptides. In contrast to other allotypes of Mamu-KIR3DL05, mmKIR3DL05x preferentially bound to Mamu-A1*00201 folded with Nef_159-167_ YY9 rather than Gag_71-79_ GY9. This difference in peptide preference mapped to six amino acids in the third D1 loop predicted to contact surfaces of the peptide-MHC class I complex. These results support a recent three-dimensional model of KIR3DL1*015 bound to HLA-A*2402 [37], and reveal a role for polymorphisms in the D1 domain in determining the selectivity of KIRs for MHC class I-bound peptides. Interestingly, mmKIR3DL05x appears to be the product of a recombination event in which exon 4 sequences coding for the D1 domain were derived from a *KIR3DS* gene; an observation that is consistent with domain shuffling as a mechanism of KIR evolution in primates [58].

Unlike previously identified ligands for human KIRs, the α1 domain of Mamu-A1*00201 contains a Bw6 motif. In contrast to Bw4, the Bw6 motif has a glycine rather than an arginine at position 83 (N_77_LRNLRG_83_). Yet, Mamu-KIR3DL05 retains a glutamate at position 285, which corresponds to glutamate 282 of KIR3DL1. Since the peptides recognized by Mamu-KIR3DL05 each contain a positively charged residue at position 6 or 8 (Gag_71-79_ GSENLKSLY, Env_788-795_ RTLLSRVY and Nef_159-167_ YTSGPGIRY), it is conceivable that glutamate 285 may form an alternative salt bridge with the peptide that accounts for the peptide-dependence of Mamu-KIR3DL05. However, a charge at this position does not appear to be sufficient for binding, since the Vif_89-97_ IW9 peptide, which also contains a lysine at position 6 (ITWYSKNFW), did not result in detectable Mamu-A1*00201 tetramer staining. While the molecular interactions underlying the binding of Mamu-KIR3DL05 to Mamu-A1*00201 remain to be fully defined, these observations offer a potential explanation for the contribution of the peptide to this interaction, and perhaps suggest a more prominent role for certain peptides in KIR recognition of other Bw6 ligands.

The extent to which KIR recognition of Bw6 ligands has been elaborated in the rhesus macaque is presently unclear. However, since this motif is retained in the MHC class I molecules of humans and macaques, the absence of human KIRs that recognize HLA-Bw6 appears to reflect the loss of receptors of this specificity during the course of human evolutionary history. While the reason for this is not understood, it may be related to the expansion of the lineage III *KIR* genes coding for KIR2DL/S receptors with a D1-D2 configuration, and a greater dependence on the regulation of NK cell activation through interactions with their HLA-C ligands.

The identification of inhibitory KIRs that bind with high avidity to a common MHC class I molecule in the rhesus macaque in complex with SIV-derived peptides suggests a potential mechanism of immune evasion. The Nef proteins of HIV-1 and SIV selectively downregulate MHC class I molecules from the surface of infected cells to evade destruction by virus-specific CD8^+^ T cells [6], [59]. However, the removal of these molecules from the cell surface increases the susceptibility of infected cells to elimination by NK cells [6]. By acquiring changes in CD8^+^ T cell epitopes that increase the binding of MHC class I ligands to inhibitory KIRs, the virus may prevent the activation of NK cells under conditions of incomplete downregulation by Nef. This possibility is supported by recent evidence that peptides can modulate NK cell activation by varying the affinity of HLA ligands for inhibitory KIRs [7]. Whereas Fadda *et al.* show that antagonistic peptides that disrupt MHC class I interactions with inhibitory KIRs leads to NK cell activation [7], our data suggests that viruses may acquire changes in epitopes that stabilize these interactions to suppress NK cell activation in a way that favors virus replication.

KIRs are also expressed on subsets of memory CD8^+^ T cells in HIV-1 infected individuals, and have been associated with a decrease in the responsiveness to TCR-dependent stimulation [44], [60]. Thus, peptides that stabilize interactions with inhibitory KIRs may also suppress CD8^+^ T cell activation. Deleterious combinations of *KIR* and *MHC class I* alleles may therefore select for changes in epitopes of HIV-1 and SIV that inhibit certain NK cell and CD8^+^ T cell responses; a scenario that may further undermine the host's ability to contain virus replication. Consistent with this hypothesis, a single nucleotide polymorphism was recently identified as a marker for two *Mamu-KIR3DL05* alleles that were more prevalent among SIV-infected rhesus macaques with high viral loads in animals [61].

The identification of Mamu-A1*00201 as a ligand for Mamu-KIR3DL05 now affords an opportunity to investigate the functional implications of KIR-MHC class I interactions. Using *KIR-* and *MHC class I*-defined animals, experiments can now be designed to examine the phenotypic changes that occur in a specific population of NK cells during the course of virus infection in a way the was previously only possible for CD8^+^ T cells. Characterization of the molecular interactions underlying the binding of Mamu-KIR3DL05 to Mamu-A1*00201 also promises to yield fundamental insights regarding the role of viral peptides in modulating KIR recognition of MHC class I ligands. The binding of Mamu-KIR3DL05 to Mamu-A1*00201 in complex with SIV peptides suggests that these interactions may be particularly important in determining the course of SIV infection.

## Materials and Methods

### Ethics statement

All of the animals used for these studies were Indian origin rhesus macaques *(Macaca mulatta)*. These animals were housed at the New England Primate Research Center (NEPRC) and were maintained in accordance with standards of the Association for Assessment and Accreditation of Laboratory Animal Care and the Harvard Medical School Animal Care and Use Committee. Animal experiments were approved by the Harvard Medical Area Standing Committee on Animals and conducted according to the principles described in the *Guide for the Care and Use of Laboratory Animals* [62].

### KIR nomenclature and Genbank accession numbers

Rhesus macaque *KIR* sequences were submitted to Genbank and to the Immuno-Polymorphism Database (www.ebi.ac.uk/ipd/kir/) [63]. Sequences that have been assigned official names are indicated with the prefix *Mamu*-*KIR*. In cases where official names have not yet been assigned, sequences are referred to using a provisional nomenclature indicated by the prefix *mmKIR*. The names and Genbank accession numbers for each of the *KIR* alleles in this study are listed in Table S1.

### Phenotypic analysis of tetramer-positive lymphocytes

Whole blood was stained with Mamu-A1*00201 tetramers folded with the SIV peptides Gag_71-79_ GY9, Env_788-795_ RY8, Env_317-325_ KM9, Nef_248-256_ LM9, Nef_159-167_ YY9, Env_296-304_ RY9, Vif_97-104_ WY8, or Vif_89-97_ IW9 (30 min, 37°C) followed by antibodies to cell type-specific markers (30 min, 20°C). Mamu-A1*00201 tetramers were obtained from David Watkins' laboratory (Wisconsin National Primate Research Center), and the quality of each tetramer lot was verified by staining CD8^+^ T lymphocytes from SIV-infected rhesus macaques. For polychromatic assays, samples were stained with anti-CD3-Pacific blue (SP34-2, BD Pharmingen), anti-CD4-AmCyan (L200, BD Pharmingen), anti-CD16-FITC (3G8, BD Pharmingen), anti-HLA-DR-PE Texas Red (Immu-257, Immunotech), anti-CD20 PE-Cy5.5 (L27, BD Pharmingen), anti-CD56 PE-Cy7 (NCAM16.2, BD Pharmingen), anti-CD8α-Alexa 700 (RPA-T8, BD Pharmingen), anti-CD14-APC-Cy7 (MphiP9, BD Pharmingen), and either anti-NKG2A-PE (Z1999, Beckman Coulter), anti-NKp46-PE (BAB21, Immunotech), anti-KIR2D-PE (NKVFS1, Miltenyi Biotec Inc.), or anti-NKG2D-PE (BAT221, Miltenyi Biotec Inc.) For four-color assays, samples were stained with anti-CD3-FITC (SP34-2, BD Pharmingen), anti-CD16-PE (3G8, BD Pharmingen), and anti-CD8α-PerCP (SK1, BD Pharmingen). Samples were treated with FACS Lysing solution (BD Biosciences) to eliminate red blood cells, washed and fixed in 2% paraformaldehyde PBS. Data was acquired using a LSRII flow cytometer (BD Biosciences) and analyzed using FlowJo 8.8.6 (Tree Star Inc.).

### Cloning and sequencing of rhesus macaque KIR alleles

Peripheral blood lymphocytes were isolated over Ficoll (Sigma) and aliquots of 2–10 million PBMC were frozen in Trizol (Invitrogen). Total RNA was extracted using the RNeasy kit (Qiagen) according to the manufacturer's instructions. KIR cDNAs were amplified by reverse transcription-polymerase chain reaction (RT-PCR) using the Superscript III One-Step RT-PCR kit (Invitrogen) with modified versions of the Ig3Up and Ig3Down primers [64]. Cycling conditions included an RT step at 55°C for 30 min, a denaturation step at 94°C for 2 min, followed by 40 cycles of denaturation (94°C for 15 sec), annealing (55°C for 30 sec) and extension (68°C for 90 sec), and a final extension step at 68°C for 5 min. PCR products were cloned into the pGEM-T Easy vector (Promega) and sequenced with T7 and SP6 sequencing primers. Sequences were analyzed using Sequencher 4.8 (Gene Codes Inc.) and MacVector 9.5.2 (MacVector Inc.) software packages. At least three identical cDNA clones were identified for each *KIR* allele.

### KIR expression and tetramer staining of transfected Jurkat cells

Rhesus macaque *KIRs* were PCR amplified from cDNA clones using primers to introduce an HA tag at the N-terminus of the D0 domain. The *KIR* cDNAs were then cloned into pCGCG, a bicistronic vector that co-expresses eGFP, in frame with an upstream sequence for the leader peptide of Mamu-KIR3DL05*008. Jurkat cells (1×10^7^ cells) were electroporated (250V, 975µF) with plasmid DNA (40 µg) in serum-free RPMI (400 µl) in a 0.4 cm cuvette (BioRad). After resting (10 min, 20°C), the cells were re-suspended in RPMI medium (9 ml) with 10% FBS and incubated overnight at 37°C, 5% CO_2_. After 22 hours, the cells were stained with APC-conjugated tetramers (30 min, 37°C), followed by PE-conjugated anti-HA PE (GG8-IF3.3, Miltenyi Biotec Inc.) (20 min, 20°C). The cells were washed and fixed in 2% paraformaldehyde PBS. At least 200,000 events were acquired using a FACSCalibur flow cytometer (BD Biosciences) and the data was analyzed using FlowJo 8.8.6.

### Mamu-KIR3DL05 genotyping

Genomic DNA was extracted from 1–2 million PBMC using the DNAeasy kit (Qiagen, Valencia, CA), and 10 ng was used as template in a 25 µl PCR reaction with forward and reverse primers (GAGACCCATGAACTTAGGCTTC & GCAGTGGGTCACTGGGGA) for amplification of a 156 bp sequence in exon 5 specific to *Mamu-KIR3DL05*. Primers specific for a conserved 300 bp region of *Mamu-DRB* were included as an internal control [48]. Cycling conditions included a denaturation step at 96°C for 2 min followed by 30 cycles of denaturation (94°C for 30 sec), annealing (63°C for 45 sec) and extension (72°C for 45 sec), and a final extension step at 72°C for 10 min. PCR products were separated on a 1% agarose gel containing ethidium bromide and visualized by UV transillumination.

### NK cell suppression by specific MHC class I molecules

PBMC (1×10^6^ cells) were stimulated for 18 hours with 721.221 cells, or with 721.221 cells expressing rhesus macaque MHC class I molecules, at a 5:1 ratio in the presence of anti-CD107a PE-Cy5 (clone H4A3, BD Pharmingen), Golgi-Stop and Golgi-plug (BD Pharmingen). The cells were then stained with APC-conjugated tetramers (30 min, 37°C), followed by anti-CD16-FITC, anti-NKG2A-PE, anti-CD8α-Alexa 700 and CD3 APC-Cy7 (20 min, 20°C). The cells were then permeabilized and stained for 30 min with anti-IFN-γ-PE-CY7 (Clone 4S.B3, BD Pharmingen). Samples were washed and fixed in 2% paraformaldehyde PBS. At least 200,000 lymphocyte events were collected using an LSRII flow cytometer, and the data was analyzed using FlowJo 8.8.6.

## Supporting Information

Figure S1 Mamu-KIR3DL07 does not bind to Mamu-A1*00201. (A) An alignment comparing the predicted amino acid sequences of the D0, D1 and D2 domains for six *Mamu-KIR3DL07* alleles. Positions of amino acid identity with the consensus sequence are indicated by a period. The shaded regions correspond to loops predicted to contact surfaces of the peptide-MHC class I complex. (B) Jurkat cells were electroporated with constructs expressing HA-tagged allotypes of Mamu-KIR3DL07 and stained the following day with APC-conjugated Gag_71-79_ GY9 or Nef_159-167_ YY9. The cells were then stained with a PE-conjugated antibody to the HA tag and analyzed by flow cytometry. Tetramer versus HA staining is shown after gating on the eGFP^+^ cell population.
(0.85 MB TIF)

Figure S2 The D1 domain of *mmKIR3DL05x* is identical to the D1 domains encoded by *Mamu-KIR3DS* alleles. The amino acid sequences of mmKIR3DL05x, Mamu-KIR3DS02*00402 and mmKIR3DHa are shown aligned to Mamu-KIR3DL05*008. Positions of amino acid identity are indicated with a period and translational stop sites are indicated with an asterisk. The shaded regions correspond to loops predicted to contact the peptide-MHC class I complex.
(0.28 MB TIF)

Figure S3 Target cells expressing Mamu-A1*00201 suppress the production of IFNγ by tetramer-positive NK cells. PBMC from two Mamu-A1*00201^+^ and two Mamu-A1*00201^–^ macaques were incubated overnight at a 5:1 effector to target cell ratio with parental 721.221 cells, or with 721.221 cells expressing individual rhesus macaque MHC class I molecules. Following stimulation, the samples were stained with Gag_71-79_ GY9 tetramer, followed by antibodies to CD3, CD8, CD16 and NKG2A. The samples were then fixed, permeabilized and stained with an IFNγ-specific monoclonal antibody. After gating on CD3-NK2GA^+^ lymphocytes, the frequency of tetramer-positive versus tetramer-negative NK cells expressing IFNγ was determined.
(0.34 MB TIF)

Table S1 Supplemental Table 1.
(0.04 MB DOC)
