# Supplementary figures and images for: KIR Polymorphisms Modulate Peptide-Dependent Binding to an MHC Class I Ligand with a Bw6 Motif
Source: PLoS Pathog. 2011 Mar 10;7(3):e1001316. doi: 10.1371/journal.ppat.1001316 (PMC3053351; doi:10.1371/journal.ppat.1001316)

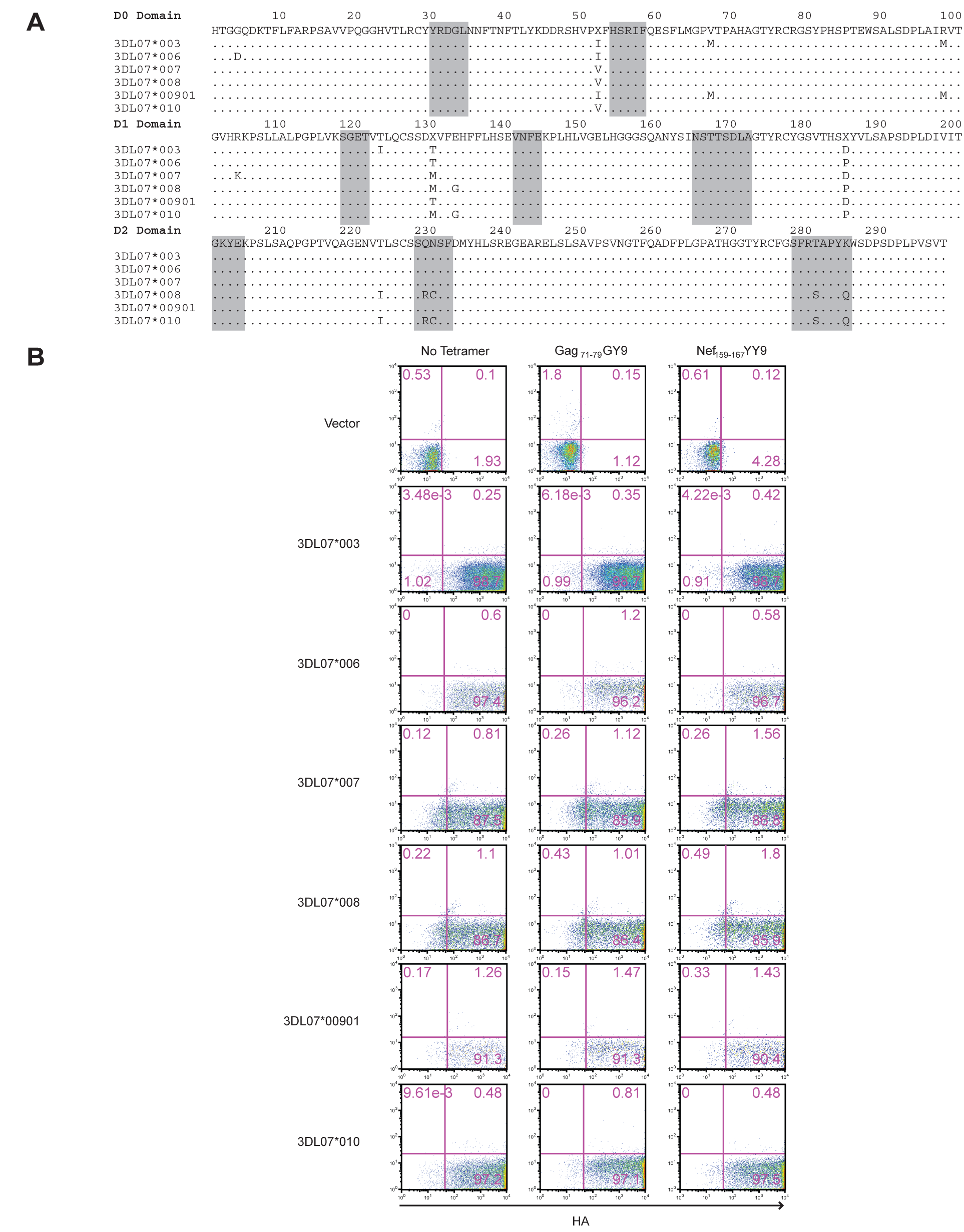

Supplement: Figure S1 — Mamu-KIR3DL07 does not bind to Mamu-A1*00201. (A) An alignment comparing the predicted amino acid sequences of the D0, D1 and D2 domains for six Mamu-KIR3DL07 alleles. Positions of amino acid identity with the consensus sequence are indicated by a period. The shaded regions correspond to loops predicted to contact surfaces of the peptide-MHC class I complex. (B) Jurkat cells were electroporated with constructs expressing HA-tagged allotypes of Mamu-KIR3DL07 and stained the following day with APC-conjugated Gag71-79 GY9 or Nef159-167 YY9. The cells were then stained with a PE-conjugated antibody to the HA tag and analyzed by flow cytometry. Tetramer versus HA staining is shown after gating on the eGFP+ cell population. (0.85 MB TIF) [file ppat.1001316.s001.tif]

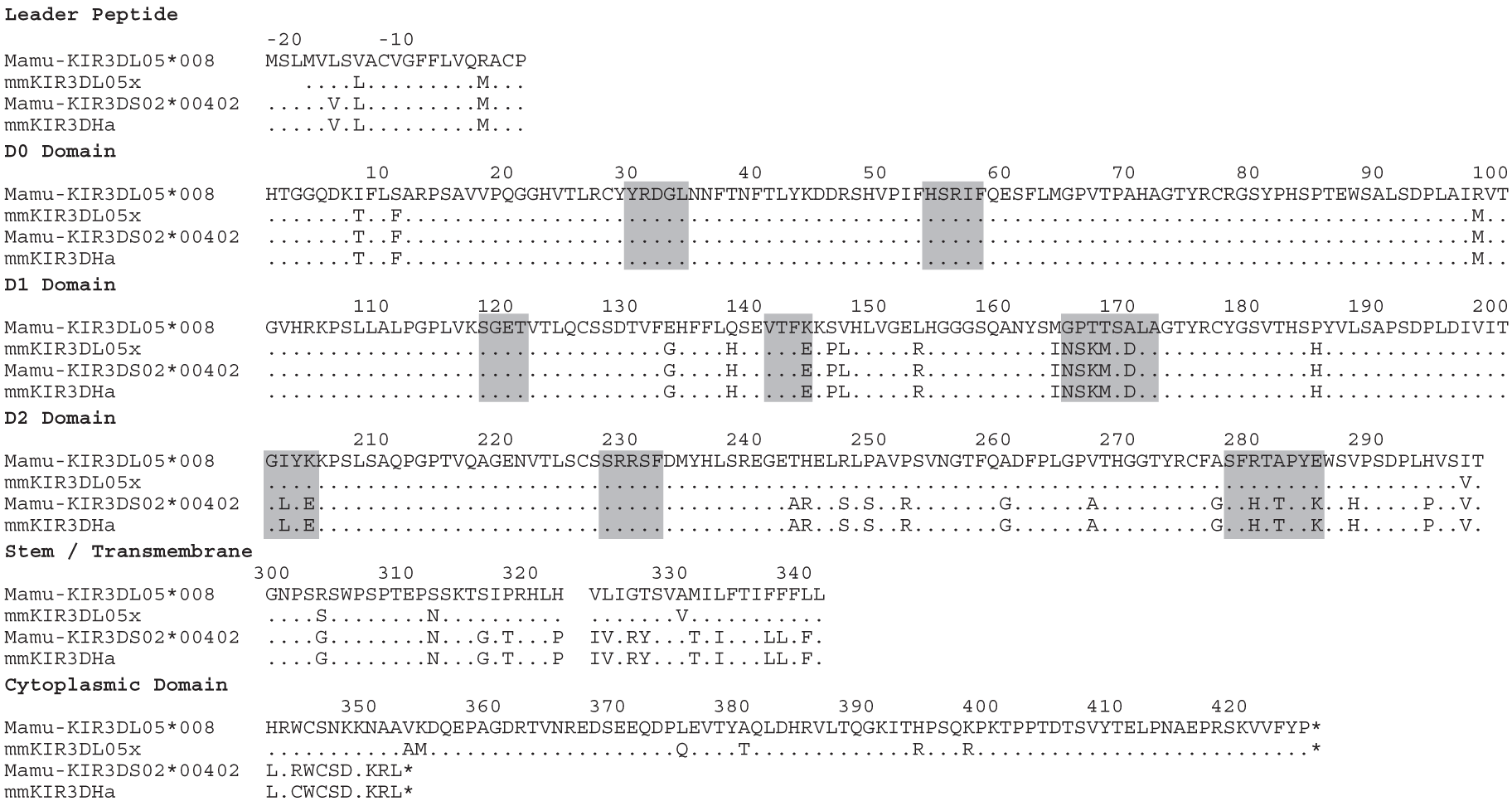

Supplement: Figure S2 — The D1 domain of mmKIR3DL05x is identical to the D1 domains encoded by Mamu-KIR3DS alleles. The amino acid sequences of mmKIR3DL05x, Mamu-KIR3DS02*00402 and mmKIR3DHa are shown aligned to Mamu-KIR3DL05*008. Positions of amino acid identity are indicated with a period and translational stop sites are indicated with an asterisk. The shaded regions correspond to loops predicted to contact the peptide-MHC class I complex. (0.28 MB TIF) [file ppat.1001316.s002.tif]

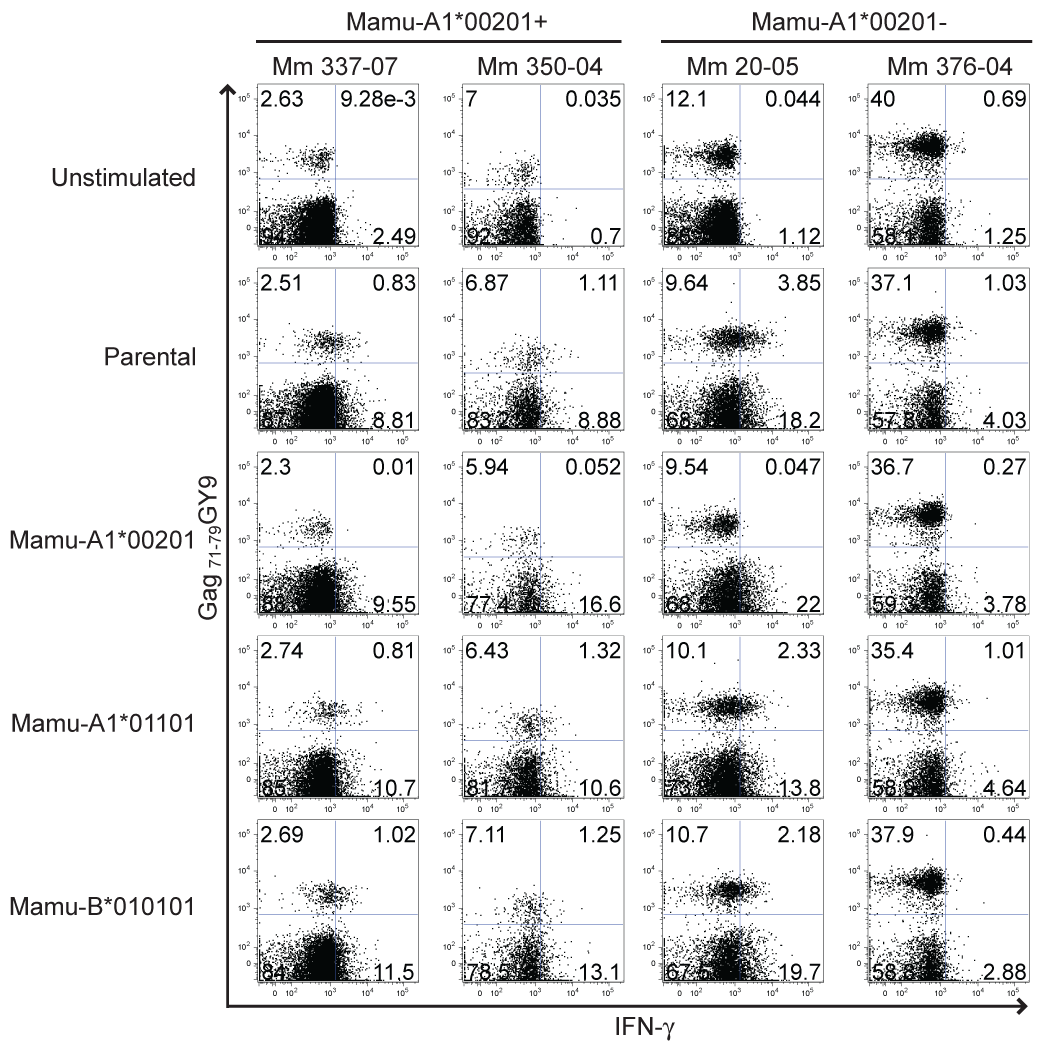

Supplement: Figure S3 — Target cells expressing Mamu-A1*00201 suppress the production of IFNγ by tetramer-positive NK cells. PBMC from two Mamu-A1*00201+ and two Mamu-A1*00201– macaques were incubated overnight at a 5:1 effector to target cell ratio with parental 721.221 cells, or with 721.221 cells expressing individual rhesus macaque MHC class I molecules. Following stimulation, the samples were stained with Gag71-79 GY9 tetramer, followed by antibodies to CD3, CD8, CD16 and NKG2A. The samples were then fixed, permeabilized and stained with an IFNγ-specific monoclonal antibody. After gating on CD3-NK2GA+ lymphocytes, the frequency of tetramer-positive versus tetramer-negative NK cells expressing IFNγ was determined. (0.34 MB TIF) [file ppat.1001316.s003.tif]
