# Supplementary material for: KIR Polymorphisms Modulate Peptide-Dependent Binding to an MHC Class I Ligand with a Bw6 Motif
Source: PLoS Pathog. 2011 Mar 10;7(3):e1001316. doi: 10.1371/journal.ppat.1001316 (PMC3053351; doi:10.1371/journal.ppat.1001316)
Supplement: Table S1 — Supplemental Table 1. (0.04 MB DOC) [file ppat.1001316.s004.doc]

**Table S1.** **Rhesus macaque KIR alleles and Genbank accession numbers**

| KIR Allele | Genbank ID | KIR Allele | Genbank ID |
| --- | --- | --- | --- |
| *Mamu-KIR3DL01*001* | GU299488 | *Mamu-KIR3DL05*001* | EU419045 |
| *Mamu-KIR3DL01*002* | GU299487 | *Mamu-KIR3DL05*003* | EU419062 |
| *Mamu-KIR3DL05*008* | GU014295 | *Mamu-KIR3DL05*004* | EU419066 |
| *Mamu-KIR3DL07*00901* | GU299489 | *Mamu-KIR3DL05*005* | EU419069 |
| *Mamu-KIR3DL10*00501* | GU014294 | *Mamu-KIR3DL05*010* | FJ562120 |
| *Mamu-KIR3DL10*00202* | GU299486 | *mmKIR3DL05x* | EU419067 |
| *Mamu-KIR3DS02*00402* | GU014296 | *Mamu-KIR3DL07*003* | EU419057 |
| *mmKIR3DHa* | GU014299 | *Mamu-KIR3DL07*006* | EU419064 |
| *mmKIR3DHb* | GU014297 | *Mamu-KIR3DL07*007* | EU419065 |
| *Mamu-KIR2DL04*00102* | GU299490 | *Mamu-KIR3DL07*008* | EU419068 |
| *Mamu-KIR2DL04*00602* | GU014298 | *Mamu-KIR3DL07*010* | EU688992 |
